# Aberrant DNMTs Promote TXNIP Upregulation and Ovarian Fibrosis in PCOS

**DOI:** 10.1096/fj.202505030R

**Published:** 2026-04-04

**Authors:** Yajing Weng, Luxi Shangguan, Qi Shen, Zhengquan Zhu, Yaling Zhang, Jingwen Zhang, Guijun Yan, Shanmei Shen, Zou Xiang, Jianguo Ruan, Yanting Wen, Daojuan Wang, Yong Wang

**Affiliations:** ^1^ Center for Reproductive Medicine and Obstetrics and Gynecology, Nanjing Drum Tower Hospital, Affiliated Hospital of Medical School, Nanjing University Nanjing China; ^2^ State Key Laboratory of Analytical Chemistry for Life Science & Jiangsu Key Laboratory of Molecular Medicine, Medical School, Nanjing University Nanjing China; ^3^ School of Medicine, Jiaxing University Jiaxing China; ^4^ Department of Endocrinology The Affiliated Nanjing Drum Tower Hospital, Medical School of Nanjing University Nanjing China; ^5^ Department of Health Technology and Informatics Hong Kong Polytechnic University Hong Kong China; ^6^ Department of Traditional Chinese Medicine The Affiliated Nanjing Drum Tower Hospital, Medical School of Nanjing University Nanjing China; ^7^ Department of Pain The Affiliated Nanjing Drum Tower Hospital, Medical School of Nanjing University Nanjing China

**Keywords:** DNA methyltransferases, granulosa cells, ovarian fibrosis, polycystic ovary syndrome, thioredoxin‐interacting protein

## Abstract

Hyperandrogenism and elevated thioredoxin‐interacting protein (TXNIP) are potential causes of infertility in women with polycystic ovary syndrome (PCOS). Epigenetic regulation of TXNIP mediates oxidative stress and inflammatory activation. However, the precise mechanisms including epigenetic regulation in PCOS are poorly understood. In this study, aberrant TXNIP in dehydroepiandrosterone (DHEA)‐induced rat PCOS ovaries and dihydrotestosterone (DHT)‐induced PCOS primary granulosa cells (GCs) coincided with a marked increase of DNA methyltransferases (DNMTs); this aberrant TXNIP triggers the release of pro‐fibrotic factors, such as collagen I, *α*‐SMA, and TGF‐*β*, from GCs. Administration of the DNMT inhibitor 5‐Aza downregulated TXNIP expression and improved the aberrant expression of the pro‐fibrotic factors in PCOS‐like ovaries and DHT‐treated GCs. Furthermore, MG132, a proteasome inhibitor, attenuated the inhibitory effect of 5‐Aza on DHT‐induced TXNIP upregulation. Our data suggest that DNMT activation, by suppressing proteasome activity, contributes to increases in TXNIP expression, resulting in ovarian fibrosis and GC dysfunction in PCOS‐like ovaries after exposure to hyperandrogenism.

## Graphical abstract

In a rat model of DHEA/DHT‐induced PCOS and in granulosa cells (GCs), upregulated DNMT1, DNMT3A, and DNMT3B inhibit TXNIP degradation, leading to TXNIP accumulation, which drives ovarian fibrosis and tissue injury.

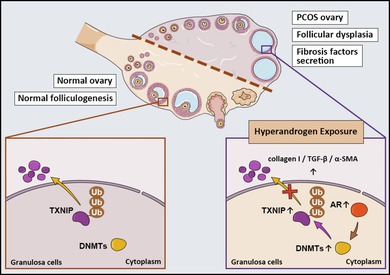

**Abbreviations**
AR: androgen receptor
BSA: bovine serum albumin
DAPI: 4′,6‐diamidino‐2‐phenylindole
DHEA: dehydroepiandrosterone
DHT: dihydrotestosterone
DNMTs: DNA methyltransferases
ECM: extracellular matrix
ER: estrogen receptors
FBS: fetal bovine serum
GCs: granulosa cells
H&E: hematoxylin and eosin
PCOS: polycystic ovary syndrome
PMSG: pregnant mare serum gonadotropin
qRT‐PCR: real‐time quantitative PCR
SD: means ± standard deviation
TRX: thioredoxin
TXNIP: thioredoxin‐interacting protein
UPS: ubiquitin‐proteasome system

## 1 Introduction

Polycystic ovary syndrome (PCOS) is a common reproductive endocrine and metabolic disorder affecting women during adolescence and reproductive years [1]. The syndrome is characterized by clinical and/or biochemical hyperandrogenism, chronic anovulation, and development of polycystic ovaries. Approximately 80% of women with anovulatory infertility have PCOS, making it the leading cause of anovulatory infertility [2, 3]. Excessive androgen, along with the ensuing ovarian fibrosis, is a major driver of anovulation in women with PCOS [4, 5]. Androgen activates transcriptional signaling by binding to the androgen receptor (AR). AR activation plays a key role in oxidative stress and mitochondrial damage [6], leading to NLRP3 inflammasome activation and pyroptosis of ovarian cells. As a consequence, ovarian fibrosis is exacerbated, ultimately resulting in ovarian dysfunction [7, 8]. However, the precise pathogenesis of ovarian fibrosis in PCOS remains to be fully elucidated.

Chronic oxidative stress and inflammation are major triggers in the development of fibrotic diseases. Thioredoxin‐interacting protein (TXNIP), an endogenous inhibitor of thioredoxin (TRX), inhibits the reductive activity of TRX through the disulfide exchange. It has been demonstrated that TXNIP is increased in fibrotic diseases, such as pulmonary fibrosis [9], hepatic fibrosis [10], and renal fibrosis [11], ultimately exacerbating fibrosis in multiple organs [12, 13]. The area of the fibrotic scar tissue after myocardial infarction is increased in TXNIP‐knockin mice, while it is ameliorated in TXNIP‐knockout mice [14]. In addition, TXNIP exacerbates renal tubular epithelial fibrosis by activating STAT3. In contrast, silencing TXNIP inhibits fibrosis and ameliorates kidney dysfunction [15]. In diabetic nephropathy, TXNIP contributes to kidney fibrosis by activating TGF‐*β* signaling and increasing fibronectin expression [16]. However, the effects of TXNIP in the occurrence and development of ovarian fibrosis are still poorly understood.

Epigenetic regulation of TXNIP plays crucial roles in the pathogenesis of various diseases, such as type 2 diabetes, diabetic kidney diseases, and renal carcinoma [17, 18, 19, 20]. DNA methylation, a crucial epigenetic modification of DNA, is regulated by DNA methyltransferases (DNMTs), including DNMT1, DNMT3A, and DNMT3B. DNMTs promote gene silencing by facilitating methylation at CpG sites, and they are implicated in diverse pathophysiological processes such as aging, tumorigenesis, immune regulation, and various cellular dynamics, including proliferation, apoptosis, and autophagy. However, the detailed regulatory patterns by which DNMTs contribute to the development of ovarian fibrosis in PCOS remain unclear.

In this study, we aim to explore the precise role and regulatory mechanisms of TXNIP in promoting ovarian fibrosis in dehydroepiandrosterone (DHEA)‐induced PCOS‐like rats. Excessive androgen causes aberrant upregulation of DNMTs, which in turn inhibits the function of the proteasome, thereby leading to the abnormal increase in TXNIP levels. Consequently, collagen deposition and ovarian dysfunction occur.

## 2 Materials and Methods

### 2.1 Animals and Experimental Protocols

Female Sprague–Dawley rats (*n* = 48, 55–65 g) were purchased from Nanjing Junke Biotechnology Corporation, China. The rats were housed, four per cage, in a specific pathogen‐free environment (Jiangsu Key Laboratory of Molecular Medicine) under a 12 h light/dark cycle at 24°C, with free access to food and water. All experimental procedures were conducted in accordance with institutional guidelines for the care and use of laboratory animals, and the study was approved by the Institutional Animal Care and Use Committee of Nanjing University (Approval No: IACUC‐D2202030).

Twenty‐one‐day‐old female SD rats were acclimated to the laboratory environment for 3 days after arrival. Daily subcutaneous injections of DHEA (6 mg/100 g body weight) were then initiated at 25 days of age and continued for 35 consecutive days to establish a PCOS‐like model, while the vehicle control group received an equal volume of soybean oil (Yuanye Biological Technology Corporation, China). For ruscogenin treatment, starting from the third week of oil or DHEA injections, rats were randomly divided into four groups (*n* = 8 in each group): (1) vehicle control: oil‐treated rats were gavaged daily with the vehicle (DMSO dissolved in corn oil) for 21 days; (2) PCOS control: DHEA‐treated rats were gavaged daily with the vehicle for 21 days; (3) RUS: oil‐treated rats were gavaged daily with ruscogenin (1 mg/kg, dissolved in DMSO and corn oil, MCE, USA) for 21 days; (4) D + *R*: DHEA‐treated rats were gavaged daily with ruscogenin (1 mg/kg) for 21 days.

For 5‐Aza treatment, starting from the fourth week of DHEA injections, the DHEA‐treated rats were randomly divided into two groups (*n* = 8 in each group): (1) DHEA + DMSO: rats were intraperitoneally injected with DMSO every 2 days for a total of seven injections; (2) DHEA + 5‐Aza: rats were intraperitoneally injected with 5‐Aza (0.6 mg/kg, MCE) every 2 days for a total of seven injections.

Following anesthesia induced by intraperitoneal injection of sodium pentobarbital (50 mg/kg), blood specimens were then drawn from the inferior vena cava and collected in disposable heparin sodium anticoagulant tubes. After standing at room temperature, the blood was centrifuged at 3000 rpm for 8 min. The upper‐layer serum was aliquoted and stored at −80°C for subsequent analysis. The ovaries were harvested and a part of the ovaries was fixed in 4% paraformaldehyde (Servicebio, China) to prepare tissue sections; the leftover ovaries and serum samples were stored at −80°C for molecular analysis.

### 2.2 Estrous Cycle Analysis

Vaginal smears were performed on the rats for 10 consecutive days starting from day 26 of modeling, at 9:30 a.m. each day. The samples were subjected to Giemsa staining and visualized under a light microscope. The proestrus stage was typified by predominantly nucleated cells; the estrus stage exhibited cornified squamous epithelial cells; the metestrus stage was marked by a combination of cornified cells and leukocytes; and the diestrus stage showed a predominance of leukocytes.

### 2.3 Isolation of Granulosa Cells (GCs)

Ovaries were harvested from 25 day old prepubertal SD rats (pre‐PCOS induction age) 48 h post‐pregnant mare serum gonadotropin (PMSG, 20 IU, Sansheng Biological Technology Corporation, China) priming to optimize GC yield and viability, a validated approach for PCOS‐relevant studies. Ovaries were collected under sterile conditions and placed in ice‐cold PBS containing 2% penicillin–streptomycin (Gibco). Ovaries were dissected free of adipose tissue and oviducts using micro‐dissecting forceps. They were then transferred to fresh DMEM‐F12 containing 2% penicillin–streptomycin. Individual follicles were isolated and placed in new wells of DMEM‐F12 with 2% penicillin–streptomycin, followed by puncture to release ovarian GCs. The cell suspension was centrifuged at 1000 rpm for 5 min, and the supernatant was discarded. The cell pellet was then resuspended in 2 mL of complete DMEM‐F12 medium and filtered through a 70 μm cell strainer. The filtered cells were collected and plated [21].

### 2.4 Cell Culture and Treatment

Primary rat GCs were cultured in DMEM‐F12 supplemented with 10% fetal bovine serum (FBS, Gibco) and 1% penicillin–streptomycin solution (Gibco) at 37°C with 5% CO_2_. To establish an in vitro hyperandrogenic PCOS‐like model, cells were treated with various concentrations (0.5, 2, and 5 μM) of dihydrotestosterone (DHT; Meilun Biological Technology Corporation, China) for 48 h. For determining the regulation of TXNIP, 5‐Aza (10 μM; MCE) or flutamide (20, 50 μM; Selleck, China) was added 24 h after the initiation of DHT treatment. MG132 (10 μM; MCE) was used to treat cells for 2 h to detect TXNIP protein degradation.

### 2.5 Hematoxylin and Eosin (H&E) and Masson Staining

All ovarian sections were deparaffinized and rehydrated. For H&E staining, the tissue sections underwent hematoxylin staining for 5 min, followed by differentiation with 1% acid‐alcohol for 30 s, rinsing briefly in distilled water, and counterstaining with eosin for 2 min. For Masson staining, the sections were sequentially incubated in Biebrich scarlet‐acid fuchsin, phosphomolybdic‐phosphotungstic acid, and aniline blue solutions for 5–10 min. After a brief rinse with distilled water, the slides were treated with 1% acetic acid for 2 min. All images were acquired using a light microscope (Leica Microsystems, Germany). Collagen fibers were visualized as blue, and the fibrotic deposition was quantified using ImageJ.

### 2.6 RNA Isolation and Real‐Time Quantitative PCR (qRT‐PCR)

Total RNA was isolated from rat ovarian tissues and GCs using TRIzol reagent (Beyotime, China), and cDNA was generated using a reverse transcription kit (Vazyme Biotech, China). qRT‐PCR was performed with the ABI Viia 7 Real‐Time PCR system (ABI, USA) using the SYBR Green PCR Master Mix (Vazyme Biotech), and the primers are shown in Table 1. The threshold cycle (Ct) values were determined and then transformed into relative quantification data using the 2^−ΔΔCt^ method. *β*‐actin served as the internal reference gene for normalization.

**TABLE 1:** qRT‐PCR primers used in the study.

Genes	Forward	Reverse
*β*‐Actin	5′‐TTCCTTCCTGGGTATGGAAT‐3′	5′‐GAGGAGCAATGATCTTGATC‐3′
TXNIP	5′‐ATCATGGCGTGGCAAGAGTC‐3′	5′‐TTTCTTGGAGCCAGGGACAC‐3′
DNMT1	5′‐TGGCAGACTCAAACCGATCC‐3′	5′‐TCCTCGTAGCCACCGAACTA‐3′
DNMT3A	5′‐TCTTCTGGGTGCTGATACTTC‐3′	5′‐GTGGTGCCAATTTTATAGGTC‐3′
DNMT3B	5′‐GTACAGGCACAGCGGAGGAT‐3′	5′‐ACAGACTTCAGAGGCAACGT‐3′

### 2.7 Western Blot

Rat ovaries and GCs were lysed with RIPA lysis buffer (Beyotime) containing 1 mM phosphatase inhibitor (MCE) and 1 mM protease inhibitor cocktail (MCE). Equal quantities of total protein were separated via 10% SDS‐PAGE, followed by transfer to polyvinylidene fluoride membranes (Merck Millipore, USA). Target proteins were then incubated with primary antibodies specific for TXNIP (1:1000; HUABIO, China), collagen I (1:1000; Bioworld Technology, USA), *β*‐catenin (1:1000; CST, USA), P‐SMAD3 (1:1000; CST), *α*‐SMA (1:1000; Bioworld Technology), TGF‐*β* (1:1000; CST), DNMT1 (1:1000; Thermo Fisher Scientific, USA), DNMT3A (1:1000; Beyotime), DNMT3B (1:1000; CST), AR (1:1000; Abcam, UK), *β*‐actin (1:20000; Fudebio, China) overnight at 4°C, after which HRP‐conjugated secondary antibodies (1:40000; Bioworld Technology) were applied. Immunoreactive bands were detected using chemiluminescent detection (Tanon, China). Quantitative densitometry was performed using ImageJ.

### 2.8 Immunohistochemistry

Ovarian tissue samples were cut into 4‐μm sections and incubated with a TXNIP‐specific primary antibody (1:100; HUABIO). The sections were then treated with goat anti‐rabbit IgG (H + L)‐HRP secondary antibody. All images were obtained using a light microscope (Leica Microsystems).

### 2.9 Immunofluorescence

GCs were seeded in 24‐well culture plates containing cell climbing slices. After adherence, the cells were fixed in 4% paraformaldehyde for 30 min at room temperature and then permeabilized with 0.3% Triton X‐100 (Beyotime). After washing with PBS three times, the cells were blocked with 3% bovine serum albumin (BSA) for 30 min at 25°C. Cells were incubated with antibodies against TXNIP (1:100), *α*‐SMA (1:100), TGF‐*β* (1:100), DNMT1 (1:50), and DNMT3A (1:100) overnight at 4°C. After washing with PBST three times, cells were incubated at 25°C for 2 h with fluorescent secondary antibodies (Beyotime). Nuclei were counterstained with 4′,6‐diamidino‐2‐phenylindole (DAPI; Beyotime) at a dilution of 1:2000 for 30 min and photographed using an Olympus laser scanning confocal microscope (FV3000, Japan).

### 2.10 Lentiviral Vector Preparation and Infection

TXNIP lentiviral particles for rats were purchased from GeneChem (China). GCs were seeded in six‐well culture plates for protein extraction and 24‐well culture plates for immunofluorescence detection. When cell confluence reached 70%, cells were incubated for 72 h with TXNIP lentiviral particles (MOI = 20) or control lentiviral particles along with HitransG P in complete DMEM‐F12 medium. The transfection efficiency was evaluated by observing the green fluorescence intensity and by confirming TXNIP protein expression via western blot.

### 2.11 RNA Interference

The TXNIP siRNA target sense oligo (5′‐GGACGUGAUUCCUGAAGAUTT‐3′) and antisense oligo (5′‐AUCUUCAGGAAUCACGUCCAT‐3′) were designed and synthesized by Keygen Biotech (China). GCs were transfected with siRNA using Lipofectamine 2000 (Invitrogen, USA) with a 72 h incubation. Next, the cells were treated with DHT (2 μM) for 48 h for various assays.

### 2.12 Statistics

All data were analyzed using GraphPad Prism 7.00. Intergroup differences were evaluated by a two‐tailed unpaired Student's *t*‐test (for two groups) or by one‐way ANOVA followed by Bonferroni's *post hoc* test (for multiple groups). The Kruskal‐Wallis test was used for data that were not normally distributed or had heterogeneous variances. Results are shown as the mean ± SD from at least three independent experiments, where n indicates the number of replicates. Statistical significance was defined as *p* ≤ 0.05.

## 3 Results

### 3.1 TXNIP Expression Is Increased in PCOS‐Like Rats and GCs Treated With Androgen

**FIGURE 1:**
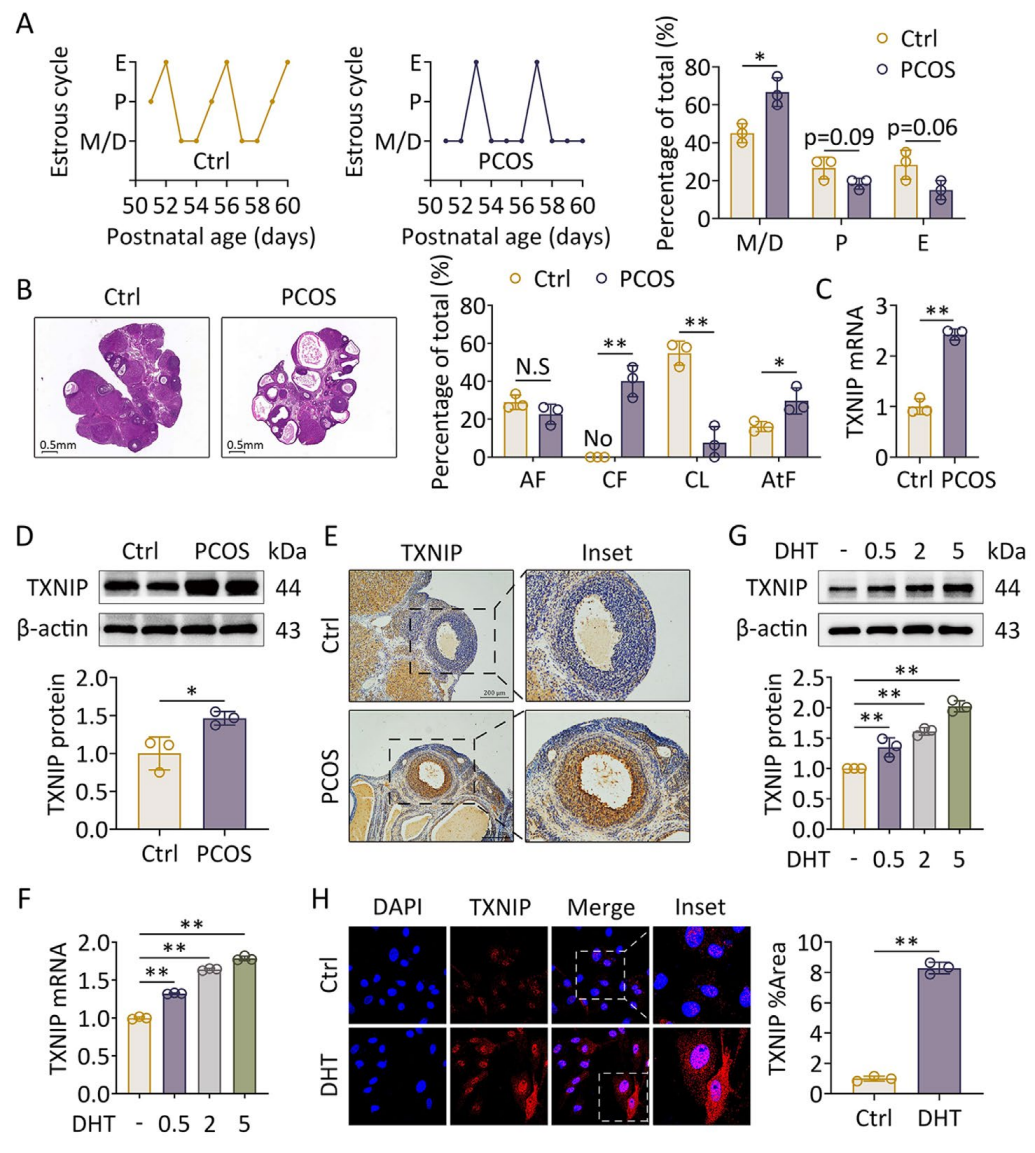
TXNIP expression is increased in PCOS‐like rats and GCs treated with androgen. Rats received DHEA and vehicle treatment (DMSO dissolved in corn oil) for induction of PCOS (*n* = 8 in each group). Primary GCs were treated with DHT (*n* = 3 in each group). (A) The estrous cycle was analyzed (left panel). The percentages of each period of the estrous cycle were quantified (*n* = 3; right panel). (B) Ovarian and follicular morphology was assessed using H&E staining (left panel). The features of follicles were analyzed (*n* = 3; right panel). (C) The mRNA levels of TXNIP in ovaries were assessed using qRT‐PCR (*n* = 3). (D) The protein levels of TXNIP in two representative ovaries of each group were assessed using western blot (upper panel). Band intensities were quantified (*n* = 3; lower panel). (E) TXNIP expression in ovaries was analyzed using immunohistochemical staining. (F) The mRNA levels of TXNIP in GCs were assessed using qRT‐PCR (*n* = 3). (G) The protein levels of TXNIP in GCs were assessed using western blot (upper panel). Band intensities were quantified (*n* = 3; lower panel). (H) Levels of TXNIP in GCs were analyzed using immunofluorescence staining (60×) (left panel). Fluorescence intensities were quantified (*n* = 3; right panel). Data are shown as the mean ± SD. *p ≤ 0.05, **p ≤ 0.01. Each treatment group was compared with the control group. AF, antral follicle; AtF, atretic follicles; CF, cystic follicles; CL, corpus luteum; Ctrl, control; D, diestrus; DHEA, dehydroepiandrosterone; DHT, dihydrotestosterone; E, estrus; M, metestrus; N.S, not significant; P, proestrus; TXNIP, thioredoxin‐interacting protein.

In this study, we established a PCOS‐like rat model using DHEA injection for 35 consecutive days. The results showed that DHEA‐treated rats exhibited disrupted estrous cycles, with prolonged durations of metestrus and diestrus (Figure 1A), reduced corpora lutea, and increased cystic follicles in the ovaries (Figure 1B). Furthermore, we examined the expression of TXNIP in the rat ovarian tissue using qRT‐PCR, western blot, and immunohistochemistry. The results showed that the expression of TXNIP in the ovaries of the PCOS‐like rats was remarkably increased compared with control at the mRNA and protein levels, especially in the rat GCs (Figure 1C–E). Next, primary GCs extracted from naive rats were treated with DHT to mimic the hyperandrogenic state in PCOS; the mRNA and protein levels of TXNIP showed a dose‐dependent increase (Figure 1F,G). Furthermore, DHT treatment prominently induced TXNIP expression (Figure 1H). Taken together, these results indicate that TXNIP is upregulated in both in vitro and in vivo hyperandrogen‐induced PCOS‐like models.

### 3.2 TXNIP Mediates Hyperandrogenism‐Induced Fibrotic Processes

**FIGURE 2:**
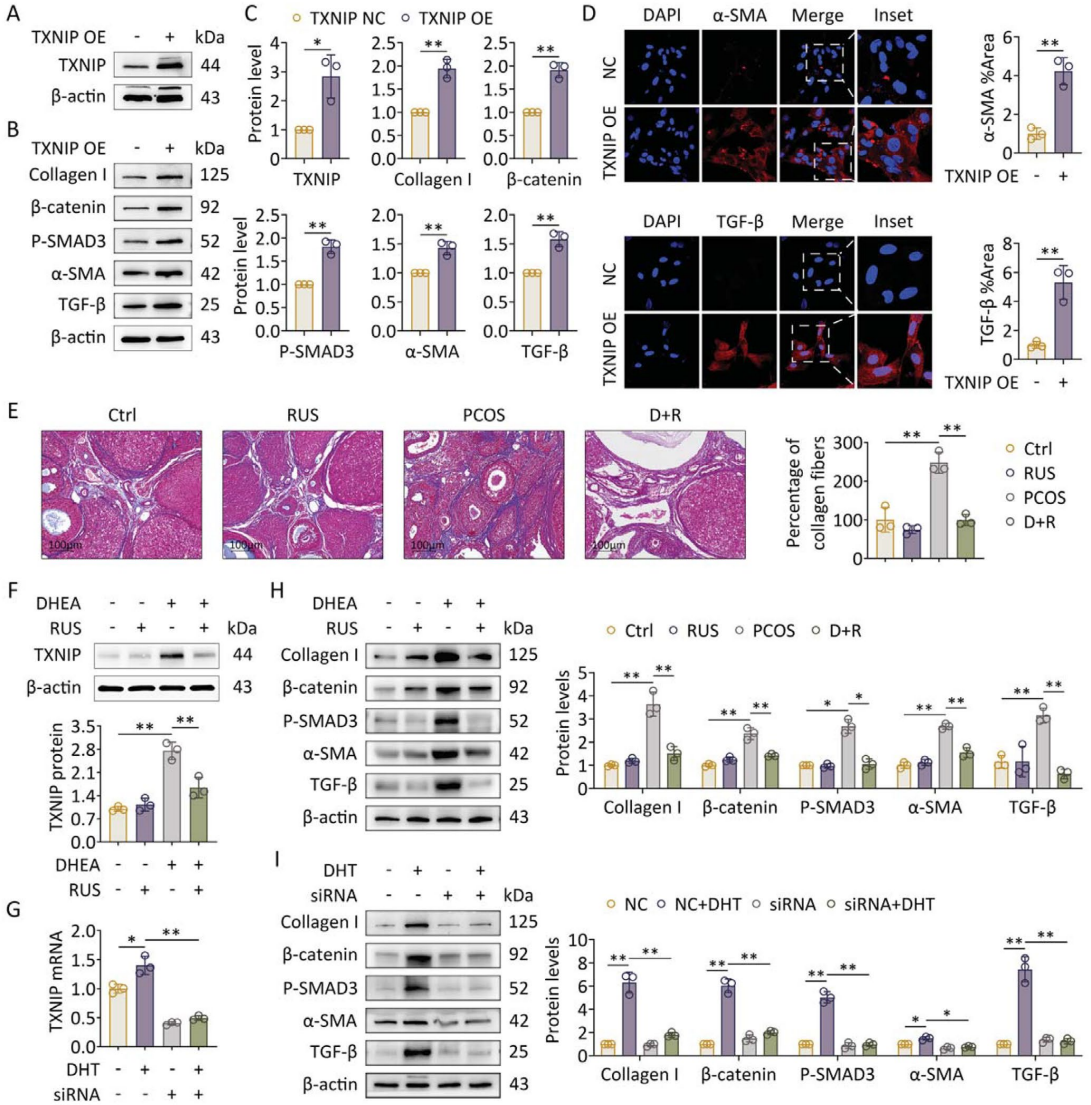
TXNIP mediates hyperandrogenism‐induced fibrotic processes. Primary GCs were transfected with lentivirus overexpressing TXNIP or siRNA followed by treatment with DHT (*n* = 3 in each group). Rats received DHEA in the presence or absence of ruscogenin, vehicle treatment (DMSO dissolved in corn oil) as control. (*n* = 8 in each group). (A) Protein levels of TXNIP in GCs were assessed using western blot. (B) Protein levels of pro‐fibrotic factors (collagen I, *β*‐catenin, P‐SMAD3, *α*‐SMA, and TGF‐*β*) in GCs were assessed using western blot. (C) Band intensities were quantified (A and B) (*n* = 3). (D) Levels of *α*‐SMA, and TGF‐*β* in GCs were analyzed using immunofluorescence staining (60×) (left panel). Fluorescence intensities were quantified (*n* = 3; right panel). (E) Collagen in ovarian slices was revealed using Masson staining (left panel). The quantification of collagen fibers per field was analyzed (*n* = 3; right panel). (F) Protein levels of TXNIP in ovaries of each group were assessed using western blot (upper panel). Band intensities were quantified (*n* = 3; lower panel). (G) mRNA levels of TXNIP in GCs were analyzed using qRT‐PCR (*n* = 3). (H) Protein levels of pro‐fibrotic factors (collagen I, *β*‐catenin, P‐SMAD3, *α*‐SMA, and TGF‐*β*) in ovaries were assessed using western blot (left panel). Band intensities were quantified (*n* = 3; right panel). (I) Protein levels of pro‐fibrotic factors (collagen I, *β*‐catenin, P‐SMAD3, *α*‐SMA, and TGF‐*β*) in GCs were assessed using western blot (*n* = 3; left panel). Band intensities were quantified (right panel). Data are shown as the mean ± SD. *p ≤ 0.05, **p ≤ 0.01. Each treatment group was compared with the control group. DHEA, dehydroepiandrosterone; DHT, dihydrotestosterone; si, siRNA; NC, negative control; OE, overexpression; RUS, ruscogenin; TXNIP, thioredoxin‐interacting protein.

It was reported that TXNIP aggravates the extent of fibrosis in several organs such as heart, kidney, liver, and lung. To further identify the connection between TXNIP and ovarian fibrosis, we transduced GCs with a lentivirus overexpressing TXNIP. Western blot assays revealed a significant upregulation of collagen I, *β*‐catenin, P‐SMAD3, *α*‐SMA, and TGF‐*β* protein expression following TXNIP overexpression (Figure 2A–C), a result that was corroborated by increased *α*‐SMA and TGF‐*β* signals detected via immunofluorescence (Figure 2D). To inhibit TXNIP expression, PCOS‐like rats were gavaged with ruscogenin, a known inhibitor of the TXNIP/NLRP3 pathway [22]. The results indicated that ruscogenin treatment significantly reduced collagen deposition in PCOS‐like rats (Figure 2E), while simultaneously downregulating TXNIP protein and mRNA levels (Figure 2F,G). This effect was accompanied by the decreased expression of fibrotic proteins, including collagen I, *β*‐catenin, P‐SMAD3, *α*‐SMA, and TGF‐*β* (Figure 2H). Consistently, knockdown of TXNIP in GCs also reversed the DHT‐induced overexpression of fibrotic factors (Figure 2I). Altogether, these results suggest that TXNIP is a key contributor to ovarian fibrosis in hyperandrogenic PCOS‐like environments.

### 3.3 Hyperandrogenism Drives DNMTs Upregulation in Ovaries

**FIGURE 3:**
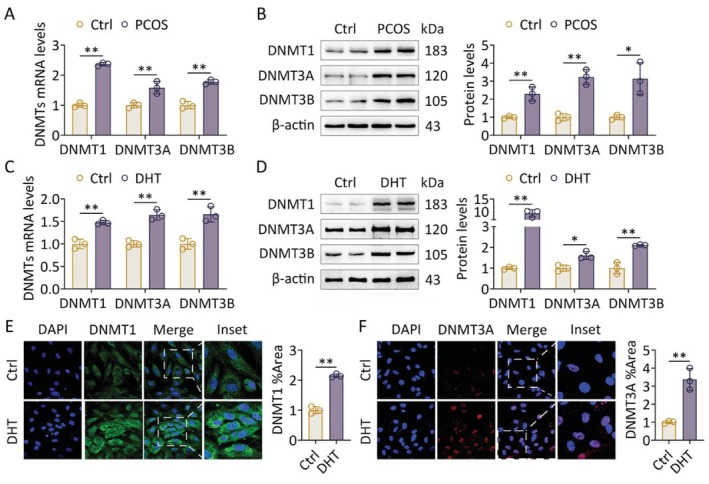
Hyperandrogenism drives DNMTs upregulation in ovaries. Rats received DHEA and vehicle treatment (DMSO dissolved in corn oil) for induction of PCOS (*n* = 8 in each group). Primary GCs were treated with DHT (*n* = 3 in each group). (A) mRNA levels of DNMTs in ovaries were analyzed using qRT‐PCR (*n* = 3). (B) Protein levels of DNMTs in two representative ovaries of each group were assessed using western blot (left panel). Band intensities were quantified (*n* = 3; right panel). (C) mRNA levels of DNMTs in GCs were analyzed using qRT‐PCR (*n* = 3). (D) Protein levels of DNMTs in GCs were assessed using western blot (left panel). Band intensities were quantified (*n* = 3; right panel). (E) Levels of DNMT1 in GCs were analyzed using immunofluorescence staining (60×) (left panel). Fluorescence intensities were quantified (*n* = 3; right panel). (F) Levels of DNMT3A in GCs were analyzed using immunofluorescence staining (60×) (left panel). Fluorescence intensities were quantified (*n* = 3; right panel). Data are shown as the mean ± SD. *p ≤ 0.05, **p ≤ 0.01. Each treatment group was compared with the control group. Ctrl, control; DHEA, dehydroepiandrosterone; DHT, dihydrotestosterone; DNMT1, DNA methyltransferase 1; DNMT3A, DNA methyltransferase 3A; DNMT3B, DNA methyltransferase 3B; DNMTs, DNA methyltransferases.

Studies have reported that DNMTs play a pivotal role in the regulation of TXNIP expression [23]. Therefore, we compared the expression levels of DNMTs in rat ovarian tissue. A significant increase in DNMTs mRNA expression was found (Figure 3A). The expression of DNMTs increased significantly in ovaries from rats treated with DHEA compared with the oil treatment (Figure 3B). Furthermore, in vitro results show that DHT upregulated DNMTs mRNA and protein expression in GCs, as determined by qRT‐PCR, western blot, and immunofluorescence (Figure 3C–F).

### 3.4 5‐Aza Treatment Inhibits Hyperandrogenism‐Induced Upregulation of TXNIP and Ovarian Fibrosis

**FIGURE 4:**
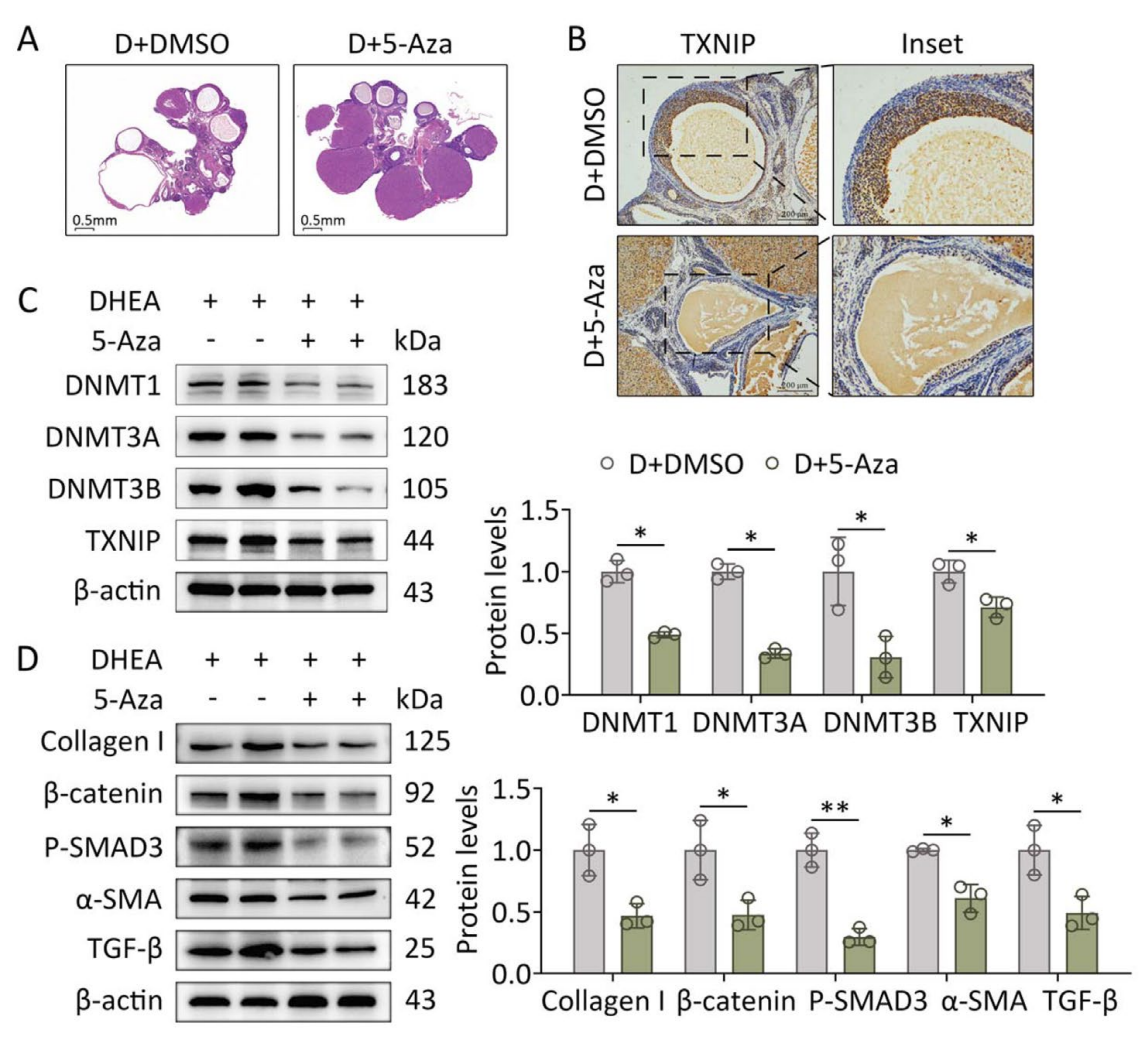
5‐Aza treatment inhibits TXNIP and ovarian fibrosis in PCOS‐like rats. Rats received DHEA in the presence or absence of 5‐Aza treatment (*n* = 8 in each group). (A) Ovarian and follicular morphology was assessed using H&E staining. (B) TXNIP expression in ovaries was analyzed using immunohistochemical staining. (C) Protein levels of DNMTs and TXNIP in two representative ovaries of each group were assessed using western blot (left panel). Band intensities were quantified (*n* = 3; right panel). (D) Protein levels of pro‐fibrotic factors (collagen I, *β*‐catenin, P‐SMAD3, *α*‐SMA, and TGF‐*β*) in two representative ovaries of each group were assessed using western blot (left panel). Band intensities were quantified (*n* = 3; right panel). Data are shown as the mean ± SD. *p ≤ 0.05, **p ≤ 0.01. Each treatment group was compared with the control group. DHEA, dehydroepiandrosterone; DNMT1, DNA methyltransferase 1; DNMT3A, DNA methyltransferase 3A; DNMT3B, DNA methyltransferase 3B; TXNIP, thioredoxin‐interacting protein.

**FIGURE 5:**
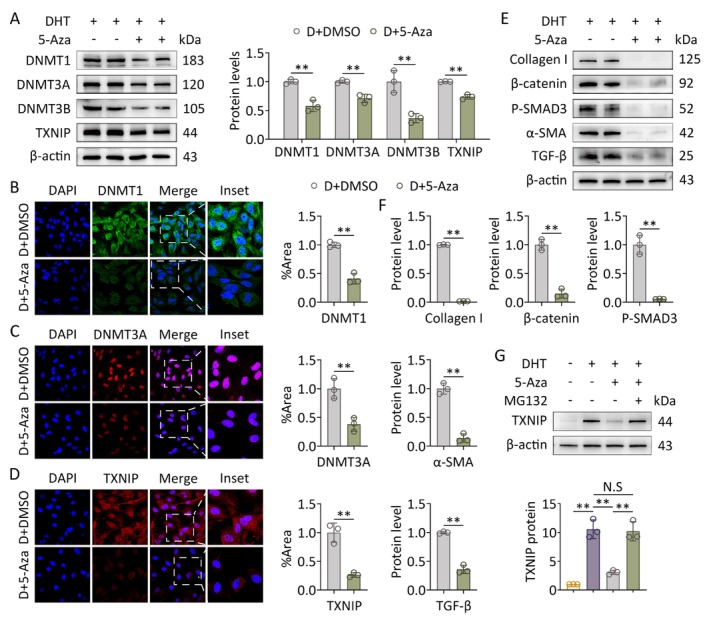
5‐Aza treatment inhibits hyperandrogenism‐induced upregulation of TXNIP and pro‐fibrotic factors in GCs. Primary GCs were treated with DHT followed by 5‐Aza and MG132 (*n* = 3 in each group). (A) Protein levels of DNMTs and TXNIP in GCs were assessed using western blot (left panel). Band intensities were quantified (*n* = 3; right panel). (B‐D) Levels of DNMT1, DNMT3A, and TXNIP in GCs were analyzed using immunofluorescence staining (60×) (left panel). Fluorescence intensities were quantified (*n* = 3; right panel). (E) Protein levels of pro‐fibrotic factors (collagen I, *β*‐catenin, P‐SMAD3, *α*‐SMA, and TGF‐*β*) in GCs were assessed using western blot. (F) Band intensities were quantified (E) (*n* = 3). (G) Protein levels of TXNIP in GCs were assessed using western blot (upper panel). Band intensities were quantified (*n* = 3; lower panel). Data are shown as the mean ± SD. *p ≤ 0.05, **p ≤ 0.01. Each treatment group was compared with the control group. DHT, dihydrotestosterone; DNMT1, DNA methyltransferase 1; DNMT3A, DNA methyltransferase 3A; DNMT3B, DNA methyltransferase 3B; N.S, not significant; TXNIP, thioredoxin‐interacting protein.

Based on our findings, abnormal activation of DNMTs was noticed in GCs of PCOS‐like rats. We further probed whether the DNMTs inhibitor 5‐Aza can inhibit TXNIP expression and ovarian fibrosis in PCOS. The PCOS‐like rats were intraperitoneally injected with 5‐Aza (0.6 mg/kg) every 2 days. After the end of the intervention, 5‐Aza treatment reduced the number and size of cystic follicles and remarkably increased the number of corpora lutea in PCOS‐like rats (Figure 4A). Immunohistochemistry showed that the positive staining of TXNIP markedly declined in GCs of the DHEA + 5‐Aza group compared to those in the DHEA group (Figure 4B). DNMTs and TXNIP expression were suppressed at the protein levels by 5‐Aza treatment in ovaries of PCOS‐like rats (Figure 4C). In addition, the expression levels of pro‐fibrotic factors also decreased significantly following 5‐Aza treatment (Figure 4D). In vitro, GCs were treated with 10 μM 5‐Aza or solvent control for 24 h with or without DHT treatment. The in vitro results followed the same trend: 5‐Aza treatment could conspicuously reverse the increase in DNMTs and TXNIP induced by hyperandrogenism (Figure 5A–D) while downregulating the mRNA and protein levels of the indicated factors related to fibrosis (Figure 5E,F). To further interrogate the mechanism of upregulated TXNIP expression by DNMTs, we treated GCs with a proteasome inhibitor MG132 following exposure to hyperandrogenism. 5‐Aza‐induced TXNIP suppression was reversed by MG132 (Figure 5G). Taken together, these results suggest that hyperandrogenism suppresses the ubiquitin‐proteasomal protein degradation of TXNIP by upregulating DNMTs, thereby increasing TXNIP expression and promoting the process of fibrosis in GCs.

### 3.5 Selective Inhibition of AR Downregulates DNMTs, TXNIP, and Pro‐Fibrotic Factors

**FIGURE 6:**
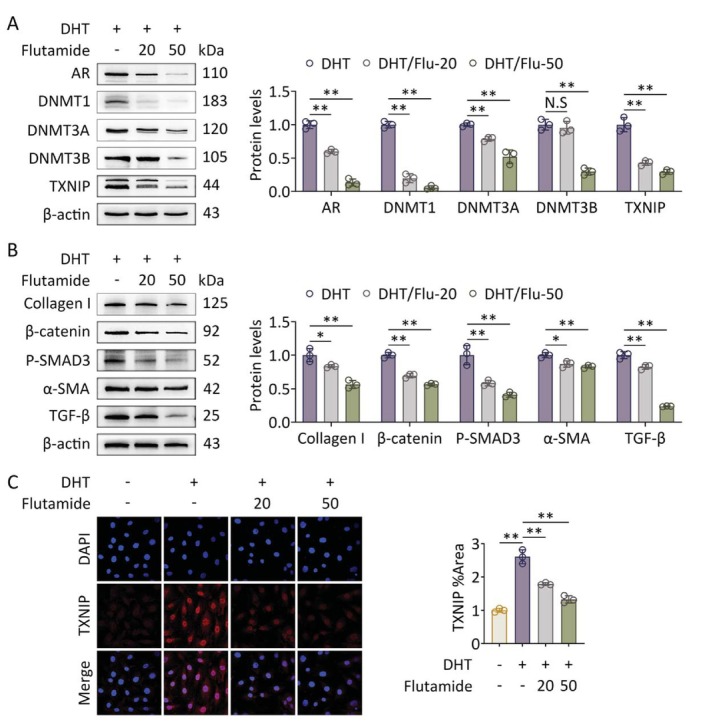
Selective inhibition of AR downregulates DNMTs, TXNIP and pro‐fibrotic factors. Primary GCs were treated with DHT followed by flutamide (*n* = 3 in each group). (A) Protein levels of AR, DNMTs, and TXNIP in GCs were assessed using western blot (left panel). Band intensities were quantified (*n* = 3; right panel). (B) Protein levels of pro‐fibrotic factors (collagen I, *β*‐catenin, P‐SMAD3, *α*‐SMA, and TGF‐*β*) in GCs were assessed using western blot (left panel). Band intensities were quantified (*n* = 3; right panel). (C) Levels of TXNIP in GCs were analyzed using immunofluorescence staining (60×) (left panel). Fluorescence intensities were quantified (*n* = 3; right panel). Data are shown as the mean ± SD. *p ≤ 0.05, **p ≤ 0.01. Each treatment group was compared with the control group. AR, androgen receptor; DHT, dihydrotestosterone; DNMT1, DNA methyltransferase 1; DNMT3A, DNA methyltransferase 3A; DNMT3B, DNA methyltransferase 3B; TXNIP, thioredoxin‐interacting protein.

Lastly, to confirm that the aforementioned pathological changes of ovarian fibrosis are directly mediated by hyperandrogenism, flutamide, an AR inhibitor, is used to block AR signaling, as it specifically antagonizes androgen action. The results showed that flutamide reduced the expression levels of AR, DNMTs, and TXNIP in a dose‐dependent manner in GCs exposed to hyperandrogenism (Figure 6A). The upregulation of collagen I, *β*‐catenin, P‐SMAD3, *α*‐SMA, and TGF‐*β* was also reversed by flutamide (Figure 6B). DHT treatment induced TXNIP expression, whereas flutamide robustly reduced DHT‐induced TXNIP levels in GCs (Figure 6C). Taken together, these results suggest that preventing AR function by blocking androgen binding to AR ameliorates fibrosis in GCs by suppressing the expression of TXNIP and pro‐fibrotic factors.

## 4 Discussion

In this study, we elucidated the precise role of aberrant TXNIP in GC dysfunction and ovarian fibrosis within the DHEA‐induced PCOS‐like rat model. We found that methyltransferases DNMT1/3A/3B inhibit the proteolysis of TXNIP by suppressing proteasome activity, leading to increased TXNIP levels and PCOS pathologies. The DNMT‐selective inhibitor 5‐Aza reversed TXNIP upregulation and improved ovarian fibrosis in PCOS‐like rats. Hence, our study provides novel targets and therapeutic strategies for PCOS.

Growing evidence strongly implicates oxidative stress as a fundamental molecular driver of PCOS. For example, mitochondrial dysfunction, redox imbalance, and elevated cellular oxidative stress lead to GC impairment, follicular dysplasia, and ultimately, ovarian tissue damage in PCOS women [24]. TXNIP, a key regulator of cellular redox homeostasis, has been previously implicated in insulin resistance [25] and GC oxidative stress [26] in PCOS. In this study, our investigation revealed an aberrant upregulation of TXNIP in PCOS ovaries; notably, pharmacologically inhibiting TXNIP with ruscogenin or genetically silencing it via siRNA significantly ameliorated ovarian fibrosis and reversed PCOS pathological alterations. These findings suggest a potential target for treating ovarian fibrosis in PCOS.

DNMTs, including DNMT1/3A/3B, inhibit gene transcription in a reversible manner and have been implicated in the pathogenesis of PCOS [27]. Previous studies have revealed that DNMT1 and DNMT3A regulate TXNIP protein stability in Schwann cells under metabolic stress, and this regulation can be blocked by the DNMT‐selective inhibitor 5‐Aza [23]. In this study, significant upregulation of DNMT1/3A/3B in PCOS ovaries impairs TXNIP protein degradation, leading to TXNIP accumulation and ovarian fibrosis, which is partially reversed by 5‐Aza. DNMT1 likely serves as the dominant isoform in GCs due to its role in S‐phase maintenance during proliferation, with DNMT3A/3B contributing de novo methylation in PCOS [28]. Isoform‐specific siRNAs or CRISPR will clarify their relative contributions in future studies. Although in vivo 5‐Aza treatment rescued PCOS‐like phenotypes, circulating levels of testosterone were not assessed. Future studies will evaluate whether TXNIP inhibition indirectly ameliorates hyperandrogenemia via restored folliculogenesis and steroidogenesis, enhancing clinical translatability.

The cellular abundance of TXNIP is tightly regulated by the ubiquitin‐proteasome system (UPS), a mechanism conserved across diverse cell types and pathophysiological contexts. TXNIP proteasomal degradation is mediated by specific E3 ubiquitin ligases and upstream signaling pathways [29, 30, 31, 32]. In metabolic diseases such as type 2 diabetes, the proteasome inhibitor MG132 blocks TXNIP degradation, thereby enhancing glucose uptake and metabolism in *β*‐cells, and exerting protective effects against *β*‐cell dysfunction and insulin resistance [33, 34, 35]. Regarding the molecular linkage between DNMT upregulation and proteasome suppression, our functional data demonstrate that proteasome inhibitor MG132 completely reverses 5‐Aza‐mediated TXNIP reduction in DHT‐treated GCs (Figure 5G), establishing proteasome‐dependent TXNIP degradation as the downstream effector. While direct DNMT‐proteasome interactions remain to be fully elucidated, emerging evidence supports multiple plausible mechanisms. For instance, the E3 ligase UHRF1 promotes DNMT1 ubiquitination and degradation, while the deubiquitinase HAUSP counteracts this process to maintain DNMT1 stability, suggesting reciprocal regulation [36]. Collectively, these findings position the DNMT‐proteasome‐TXNIP axis as a novel therapeutic target for PCOS ovarian fibrosis.

The DHEA‐induced model, while valuable for recapitulating hyperandrogenemia, irregular cycles, and metabolic features of PCOS, exhibits more pronounced cystic degeneration than typically observed in human PCOS ovaries, which primarily feature multifollicular morphology with excessive small‐to‐medium antral follicles and heightened atresia [37]. This limitation is acknowledged, yet the model's high reproducibility supports its utility for therapeutic testing. In addition, chronic subcutaneous DHEA administration in female rats leads to elevated circulating levels of testosterone (via direct conversion) and estradiol (via peripheral aromatization of DHEA/testosterone), thereby activating both AR and estrogen receptors (ER) systemically, mimicking PCOS‐like multi‐hormonal dysregulation. In contrast, our in vitro experiments using DHT—a non‐aromatizable androgen—on isolated GCs isolate AR‐mediated effects, excluding ER influence or metabolic conversion, to dissect direct GC responses [38]. The complementary use of systemic DHEA (dual AR/ER activation) and targeted DHT (AR‐specific) elucidates both holistic PCOS phenotypes and cell‐autonomous mechanisms, despite their differing hyperandrogenic milieus.

## 5 Conclusions

In summary, this study confirmed that in DHEA‐ and DHT‐induced PCOS‐like rats and GCs, the aberrant increase of DNMT1/3A/3B mediated the inhibition of TXNIP degradation, leading to TXNIP upregulation and subsequent ovarian fibrosis and damage in PCOS. These findings provide a theoretical rationale and potential therapeutic targets for the clinical study and treatment of PCOS.

## Author Contributions

Y.W. (Yajing Weng): writing – review and editing, writing – original draft, methodology, investigation, conceptualization. L.S.: methodology, investigation, conceptualization. Q.S.: software, investigation. Z.Z. and Y.Z.: methodology, investigation. J.Z. and S.S.: visualization, data curation. G.Y. and Z.X.: writing – review and editing, supervision. Y.W. (Yong Wang) and D.W.: writing – review and editing, supervision, project administration, funding acquisition, conceptualization. Y.W. (Yanting Wen) and J.R.: validation, supervision, project administration, conceptualization. All authors reviewed the manuscript.

## Funding

This research was supported by the National Natural Science Foundation of China (82471675 and 81971346) and the Three‐Year Action Plan for Further Accelerating the Inheritance, Innovation and Development of Traditional Chinese Medicine (TCM) in Major and Intractable Diseases of the Shanghai Municipal Health Commission (2‐1‐6).

## Ethics Statement

All animal experiments were conducted according to the principles and guidelines of the Institutional Animal Care and approved by the Institutional Research Animal Committee of Nanjing University. Informed consent was obtained from all individual participants included in the study.

## Conflicts of Interest

The authors declare no conflicts of interest.

## Data Availability

Data will be made available on request.
